# Personalized vaccine repurposing for AD prevention: Genotype‐specific protective association of the shingles vaccine with Alzheimer's disease

**DOI:** 10.1002/alz70860_104190

**Published:** 2025-12-23

**Authors:** Svetlana Ukraintseva, Hongzhe Duan, Rachel Holmes, Deqing Wu, Arseniy Yashkin, Igor Akushevich, Konstantin Arbeev, Anatoliy Yashin

**Affiliations:** ^1^ Duke University, Durham, NC, USA

## Abstract

**Background:**

Our earlier study using Medicare‐linked data found that vaccination against shingles was associated with ∼20% reduced risk of Alzheimer's disease (AD) later in life. Here, we investigated genotype‐specific associations of the shingles vaccine with the odds of AD in the Health and Retirement Study (HRS) and the UK Biobank (UKB).

**Method:**

The study sample included genotyped HRS survey (*N* = 9,188) and UKB primary care (*N* = 66,748) participants who survived past age 75. Logistic regression models were applied to assess the association between vaccination against shingles and the later onset of AD. The vaccination variable was defined as: 0 ‐ did not receive a shingles vaccine; 1 ‐ received at least one vaccine at ages 65‐75. The binary AD outcome was defined as: 0 ‐ AD onset after age 75; 1 ‐ no AD at any age. The analysis was stratified by carrier and non‐carrier status of the rs6859 (A) allele, a risk factor for AD in the NECTIN2 gene involved in vulnerability to infections. Observed covariates included education, smoking, race, and (when not stratified) sex.

**Result:**

Shingles vaccine received at ages 65–75 was associated with significantly lower odds of AD onset after age 75 (OR=0.55, *p*‐value=0.021) in an unstratified HRS sample. After stratification, the AD‐protective association became stronger in carriers of the rs6859 (A) allele (OR=0.44, *p*‐value=0.016), especially in females (OR=0.28, *p*‐value=0.018). In non‐carriers, the association between the shingles vaccine and AD became non‐significant (OR=0.90, *p*‐value=0.81). Similar associations of the shingles vaccine with AD were found in the UKB analysis. Lower odds of AD were observed if the vaccine was received three or more years versus two or less years before age 75 (OR: 0.39 vs. 0.61 in the total UKB sample; 0.35 vs. 0.58 in rs6859 (A) carriers; and 0.54 vs. 0.71 in non‐carriers).

**Conclusion:**

Vaccination against shingles before age 75 is associated with lower odds of AD later in life, especially in female carriers of the rs6859 (A) allele, a genetic risk factor for AD. Vaccination at a younger age may provide more protection against AD. These results support personalized repurposing of the shingles vaccine for AD prevention.

